# Shade Inhibits Leaf Size by Controlling Cell Proliferation and Enlargement in Soybean

**DOI:** 10.1038/s41598-017-10026-5

**Published:** 2017-08-23

**Authors:** Yushan Wu, Wanzhuo Gong, Wenyu Yang

**Affiliations:** 1 College of Agronomy, Sichuan Agricultural University, Chengdu, 611130 P.R. China; 2 Key Laboratory of Crop Ecophysiology and Farming System in Southwest, Ministry of Agriculture, Chengdu, 611130 P.R. China; 3 Sichuan Engineering Research Center for Crop Strip Intercropping System, Chengdu, 611130 PR China; 4 Characteristic Crops Research Institute, Chongqing Academy of Agricultural Sciences, Chongqing, 402160 P.R. China

## Abstract

To gain more insight into the physiological function of shade and how shade affects leaf size, we investigated the growth, leaf anatomical structure, hormones and genes expressions in soybean. Soybean seeds were sown in plastic pots and were allowed to germinate and grow for 30 days under shade or full sunlight conditions. Shade treated plants showed significantly increase on stem length and petiole length, and decrease on stem diameters, shoot biomass and its partition to leaf also were significantly lower than that in full sunlight. Smaller and thinner on shade treated leaves than corresponding leaves on full sunlight plants. The decreased leaf size caused by shade was largely attributable to cell proliferation in young leaves and both cell proliferation and enlargement in old leaves. Shade induced the expression of a set of genes related to cell proliferation and/or enlargement, but depended on the developmental stage of leaf. Shade significantly increased the auxin and gibberellin content, and significantly decreased the cytokinin content in young, middle and old leaves. Taken together, these results indicated that shade inhibited leaf size by controlling cell proliferation and enlargement, auxin, gibberellin and cytokinin may play important roles in this process.

## Introduction

Intercropping is practiced widely by smallholder farmers across the world^1, 2^. But shade environment by taller crops usually limited the growth of shorter crops in intercropping^3, 4^. In the natural environment, all plants are shaded to some degree by surrounding plants or themselves during their lifecycle^5^. Shade condition is mainly caused by two kinds of signaling factors, they are low proportion of red light to far red light (R: FR) and low photosynthetically active radiation (PAR)^6, 7^. When plants exposed to shade, they showed two different strategies: shade tolerance and shade avoidance^8, 9^. shade tolerance responses optimize light capture and utilization, including increases of chlorophyll content, specific leaf area, photosystem II: I ratio, and decrease of chlorophyll a:b ratio, all of which contribute to carbon gain in the leaf ^5^. Shade avoidance are induced by the signaling of low PAR and low R:FR, maximize light capture by increasing stem length and positioning the leaves out of the shade via photoreceptor signaling networks^6, 7, 10, 11^.

Leaf is the most important organ to gain carbon in shade condition. Leaf morphology, anatomy, physiology and biochemistry attribute to many shade tolerance features^5^. For shade tolerance plants, leaf area is an important feature for light capture and harvesting. In order to get more opportunities for light capture and harvesting in shade conditions, plants generally accumulate more chlorophyll content per unit mass and decrease leaf dry matter per unit area^12–15^. At whole plant level, we also found that shade decreased the absolute leaf area^5^, there was a positively relationship between shade tolerance and leaf area per plant dry matter, especially for smaller plants^16^. On other side, shade avoidance response also includes the characteristics of inhibited leaf area^17^.

Cell number and cell size are the two main factors that determine the leaf size. But the leaf area is not a simple sum of cell number and cell size, it’s under the co-ordination of them^18–20^. During the whole primordium, cell division occurs and generates new cells, the size of cells remains relatively constant and small, when cell division is finished, leaf growth is largely depends on cell expansion, which will result in an enlargement in cell size^18^. Recently, a report demonstrated that across all species and organs, cell number rather than cell size determines the final size of plant organs^21^. In shade environment, some studies found that shade reduced the number of leaves, total leaf area and individual leaf areas of Arabidopsis^22, 23^. Meanwhile, it has been reported that cell expansion is more important than cell division in leaf growth under low photosynthetically active radiation conditions^17^, but other study pointed that cell number, not cell size, contributes to the reduced leaf size of plants grown in low R/FR relative to high R/FR^24^. In general, the past studies suggested that leaf area was finally determined by cell division in young phase and cell expansion in old phases. As the recent advances made by molecular studies, many genes involves in leaf development and growth had been found^18, 25^. These findings gave an opportunity to investigate shade effects on leaf growth at molecular level.

Soybean (*Glycine max* L.) is one of major crops produced and consumed for protein and oil throughout the world. It is usually planted in intercropping systems and expressed shade avoidance^26, 27^. We have found that soybean leaves became smaller when they grown under shade condition in intercropping systems, and plant hormones might be involved in inhibition on leaf size under shade as suggested by transcriptome analysis^8, 28^. But it remains uncertain how shade effect the leaflet size of trifoliate in soybean. To gain more understanding of the effects of shade on leaf size, the objectives of this study were: 1) to investigate the morphology and cellular mechanisms of leaf growth regulated by shade in soybean. 2) to quantify the expression levels of twenty genes related to cell proliferation and/or cell enlargement in response to shade. 3) to determine the variations of auxin, cytokinin, gibberellin and brassinolide contents.

## Results

### Shade affects the morphology of soybean

**Figure 1:**
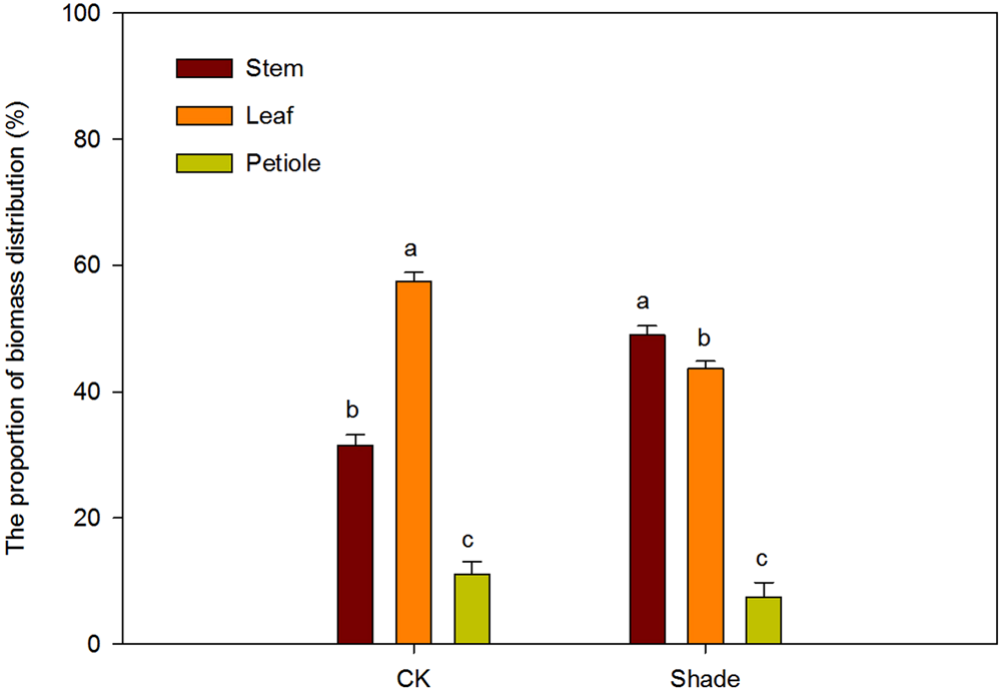
The proportion of biomass distribution of soybean planted in shade and full sunlight (CK). Values are means ± SD (n = 4). Statistical significance assessed by Duncan’s t-test. Lowercase letters indicates significant at 0.05 probability level.

**Figure 2:**
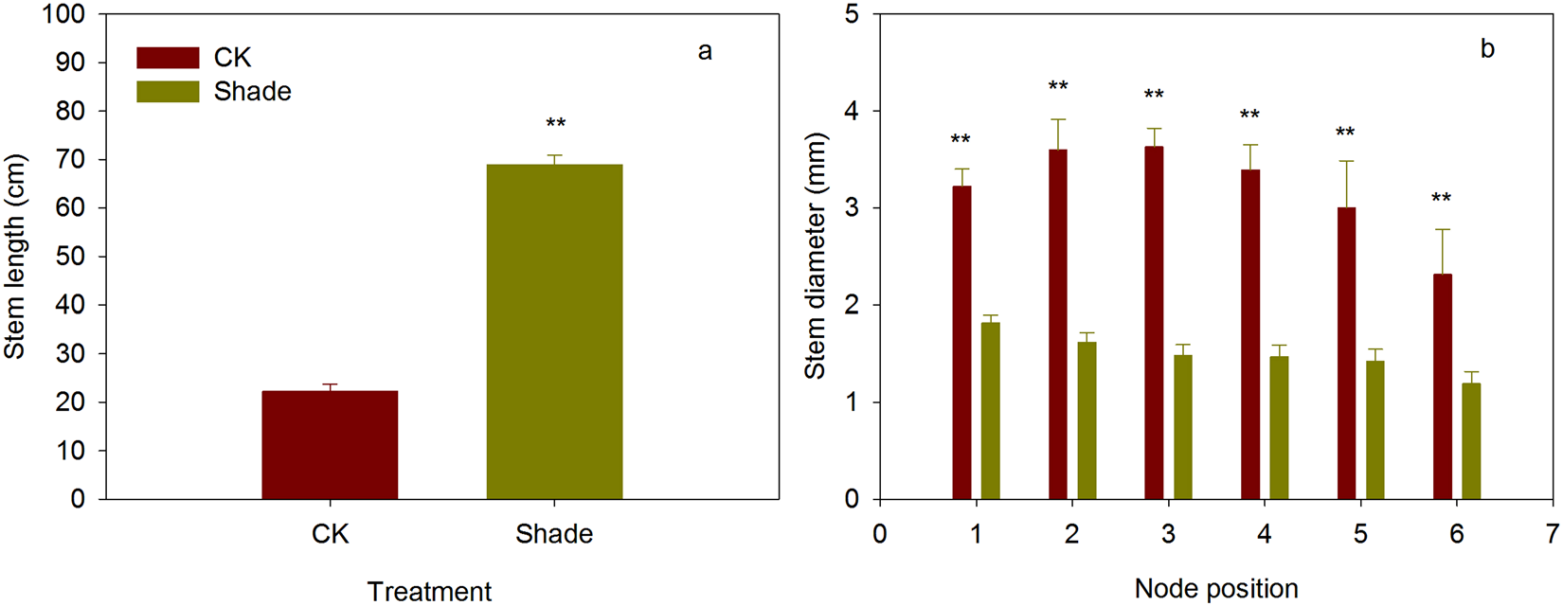
Stem length (**a**) and stem diameter (**b**) of soybean planted in shade and full sunlight (CK). Values are means ± SD (n = 4). Statistical significance assessed by Duncan’s t-test. ** indicates significant at 0.01 probability level, respectively.

Shoot biomass in shade was significantly lower than that in full sunlight (Supplementary Fig. S1). In full sunlight (CK), the proportion of biomass distribution to leaf showed significantly higher than to petiole and to stem. However, under shade, the proportion of biomass distribution to leaf was lower than to stem (Fig. 1). In addition, soybean showed significantly stem elongation response under shade, under full sunlight, stem length was 22.2 cm. Under shade, stem length increased significantly to 68.9 cm, representing 210.3% of stem elongation (Fig. 2a). However, the stem diameters of node 1 to 6 were significantly lower (0.40–0.56 times) in shade plants than in full sunlight plants in the corresponding node positions (Fig. 2b).

### Shade inhibited leaf area and leaf size

**Figure 3:**
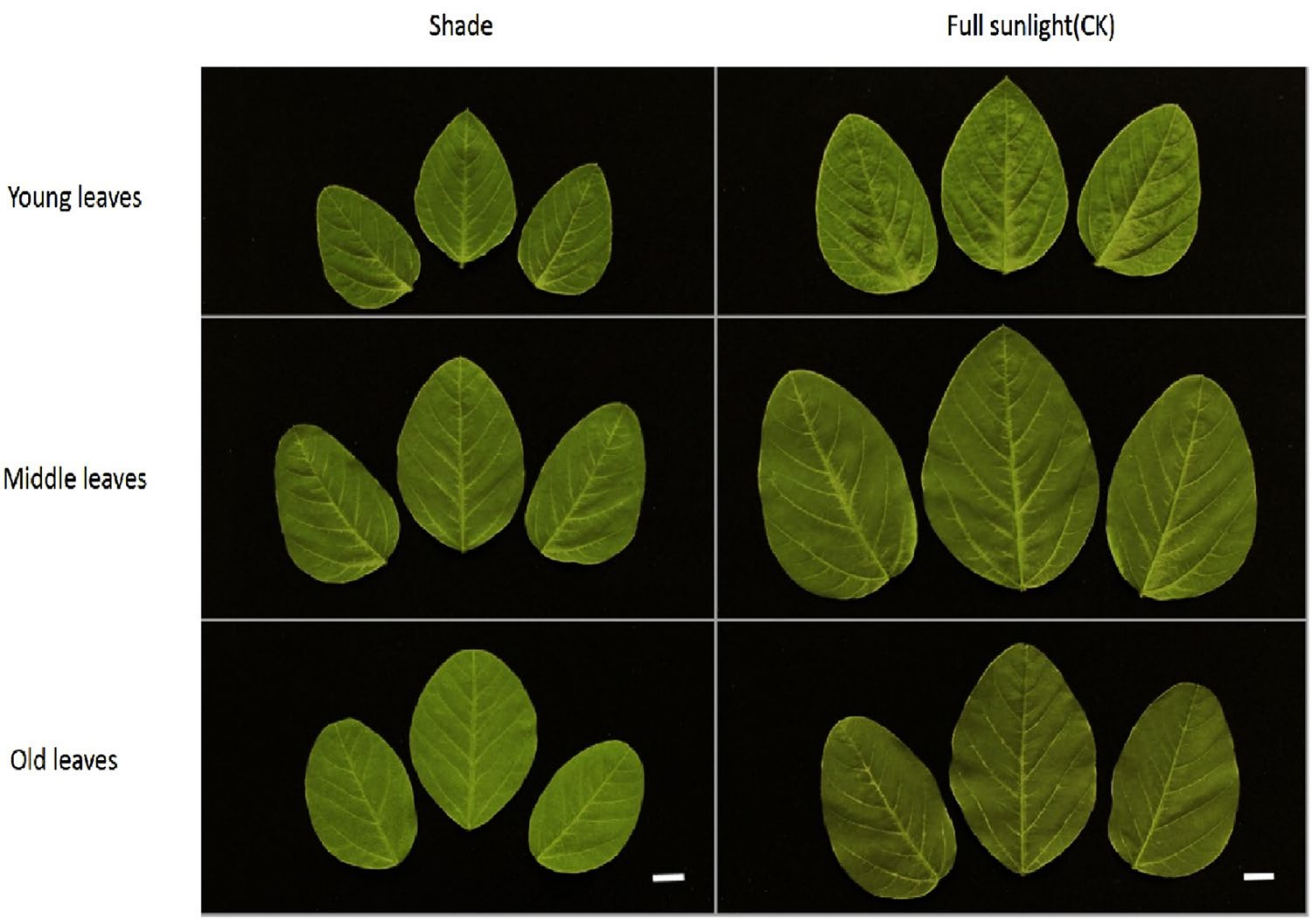
Leaves of soybean grown under shade and full sunlight (CK) treatment at 30 days after planting. Bars, 1 cm.

In shade treatment and full sunlight control, leaves 1 were fully expanded mature (old), leaves 2 were incomplete maturation (middle), leaves 3 were the youngest expanding (young) (Fig. 3). Soybean showed that the total leaf area in shade were significantly smaller than full sunlight control plants (Table 1). The areas of young, middle and old leaves were smaller (0.7–0.8 times) in shade plants than in full sunlight plants (Table 1). As showed in Table 1, the leaf size in shade were significantly smaller than sunlight control plants in young, middle and old. In addition, petiole length were significantly higher in shade than in full sunlight in the corresponding leaf positions (Table 1).

**Table 1:** Leaf area, leaflet size and petiole of soybean planted in shade and full sunlight (CK). Data are means ± SD (n = 4).

Treatment	Leaf area(cm^2^)				leaflet size (cm^2^)			Petiole length (cm)		
	Young	Middle	Old	Total area	Young	Middle	Old	Young	Middle	Old
Shade	40.93 ± 2.22b	66.88 ± 3.51b	58.88 ± 4.77b	199.65 ± 4.52b	13.64 ± 1.17b	22.29 ± 1.83b	19.63 ± 2.02b	6.72 ± 0.61a	12.78 ± 0.44a	11.43 ± 0.59a
CK	55.60 ± 2.14a	83.97 ± 4.76a	73.60 ± 5.06a	239.73 ± 4.96a	18.53 ± 1.45a	27.98 ± 2.33a	24.53 ± 2.18a	5.58 ± 0.35b	11.02 ± 0.62b	10.67 ± 0.38b

Statistical significance assessed by Duncan’s t-test. Values followed by different letters in the same column are significantly different at the 0.05 probability level (p < 0.05).

### Shade inhibited leaf cell number and cell size

**Figure 4:**
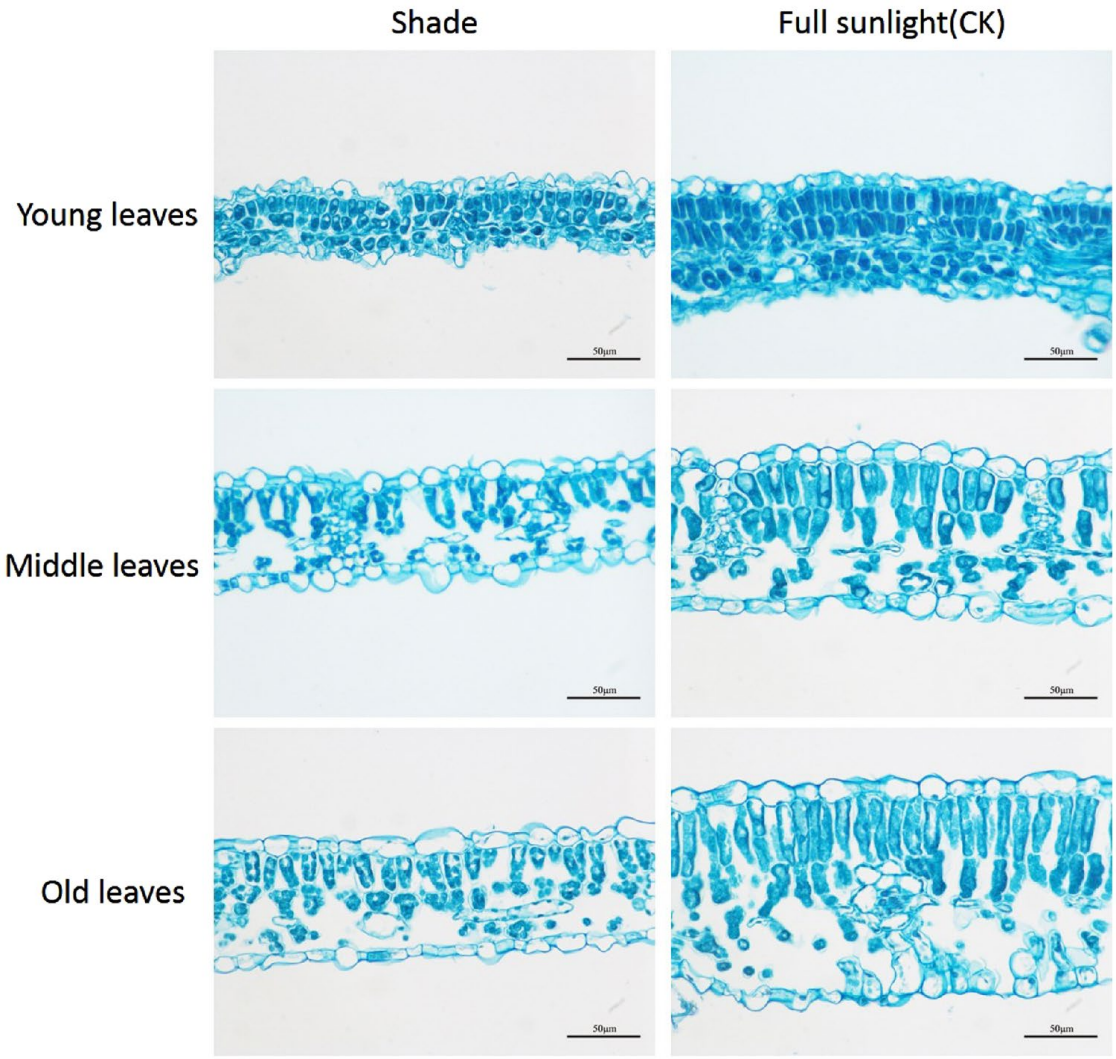
Transverse sections and microscope views of leaves of 30 days soybean plants grown in shade and full sunlight (CK). The transverse sections reveal a region between the midvein and the leaf margin. Bars, 50 µm.

**Figure 5:**
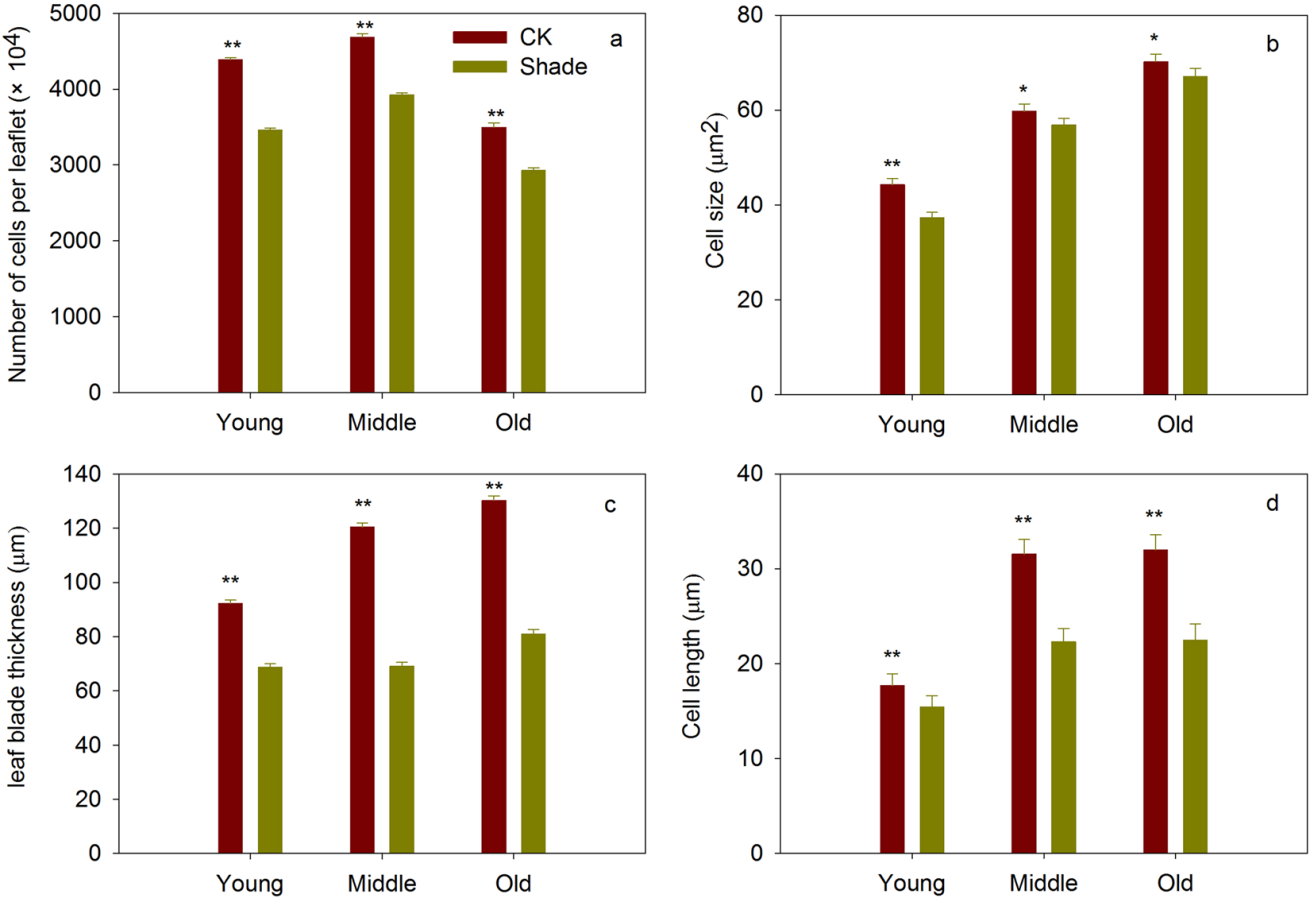
Number of cells per leaflet (**a**), cell size (**b**), leaf blade thickness (**c**) and cell length (**d**) of soybean planted in shade and full sunlight (CK). Values are means ± SD (n = 3). Statistical significance assessed by Duncan’s t-test. * and ** indicates significant at 0.05 and 0.01 probability level, respectively.

The numbers and sizes of palisade cells in young, middle and old leaves in shade plants and full sunlight plants were shown in Figs 4 and 5. Shade treatment showed significantly decreased cell numbers and cell size in young, middle, old leaves. The shade induced change in cell number (CCN: 0.79) of young leaves was significantly smaller than those (0.84) of old leaves. Meanwhile, the shade induced change in cell size (CCS: 0.84) of young leaves also appeared to be significantly smaller than that (0.96) of old leaves. In addition, in young, middle and old leaves, leaf blade thickness and cell length in shade were significantly decreased (Fig. 5c,d).

### Expression of cell proliferation and/or enlargement genes in response to shade

**Figure 6:**
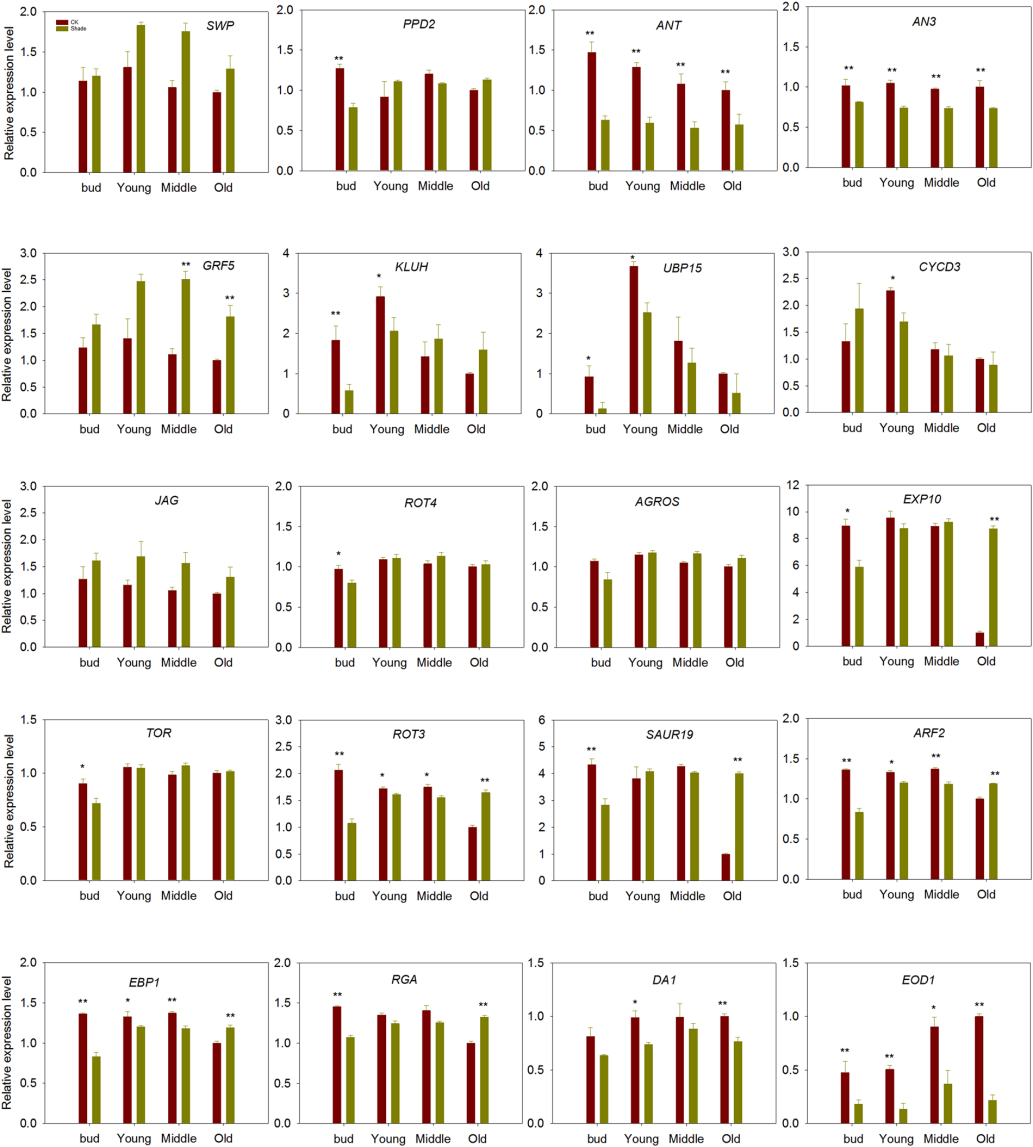
Expression levels of 20 cell proliferation and/or enlargement genes in young, middle and old leaves of 30 days old soybean plants grown in shade or full sunlight (CK). Expression levels are normalized with respect to the housekeeping gene ACT and are displayed relative to a value of unity for old leaves in full sunlight. Values are mean ± SD (n = 6). Statistical significance assessed by Duncan’s t-test. * and ** indicates significant at 0.05 and 0.01 probability level, respectively.

After the blast against Arabidposis, soybean homologues were selected to measure their expression levels in this study^18, 29, 30^. Totally, nine genes involves in cell proliferation (*ANT*, *AN3*, *GRF5*, *KLUH*, *UBP15*, *CYCD3*, *JAG*, *ROT4* and *ARGOS*), four genes involves in cell expansion (*EXP10*, *TOR*, *ROT3* and *SAUR19*), five genes involves in both cell proliferation and expansion (*ARF2*, *EBP1*, *RGA*, *DA1*and *EOD1*), and other two genes involves in determining primordium size (*SWP*) and meristemoid division (*PPD2*) were measured (Fig. 6).

In axillary bud, one negative regulator for meristemoid division: *PPD2*, five negative regulator for cell proliferation: *ANT*, *AN3*, *KLUH*, *UBP15, ROT4*, four negative regulator for cell enlargement: *EXP10*, *TOR*, *ROT3*, *SAUR19*, four negative regulators for both cell proliferation and cell enlargement: *ARF2*, *EPB1*, *RGA* and *EOD1* were significantly down-regulated in shade-treated plants compared with the controls (Fig. 6).

In young leaves, five negative regulators for cell proliferation: *ANT*, *AN3*, *KLUH*, *UBP15*, *CYCD3*, one negative regulators for enlargement: ROT3, and four negative regulators for both cell proliferation and enlargement: *ARF2*, *EBP1*, *DA1*, *EOD1* were significantly down-regulated in shade-treated plants compared with the controls (Fig. 6).

In middle leaves, two negative regulator for cell proliferation: *ANT*, *AN3*, one negative regulator for cell enlargement: *ROT3*, three negative regulator for both cell proliferation and cell enlargement: *ARF2*, *EBP1*, *EOD1*were significantly down-regulated in shade plants compared to full sunlight plants (Fig. 6).

In old leaves, two negative regulators for cell proliferation: *ANT* and *AN3* were down-regulated, three positive regulator for cell enlargement: *EXP10*, *ROT3*, *SAUR19* and three positive regulator of both cell proliferation and cell enlargement. *ARF2*, *EBP1*, *RGA* were up regulated in shade plants compared to full sunlight plants (Fig. 6).

### Shade affects the hormones content

**Figure 7:**
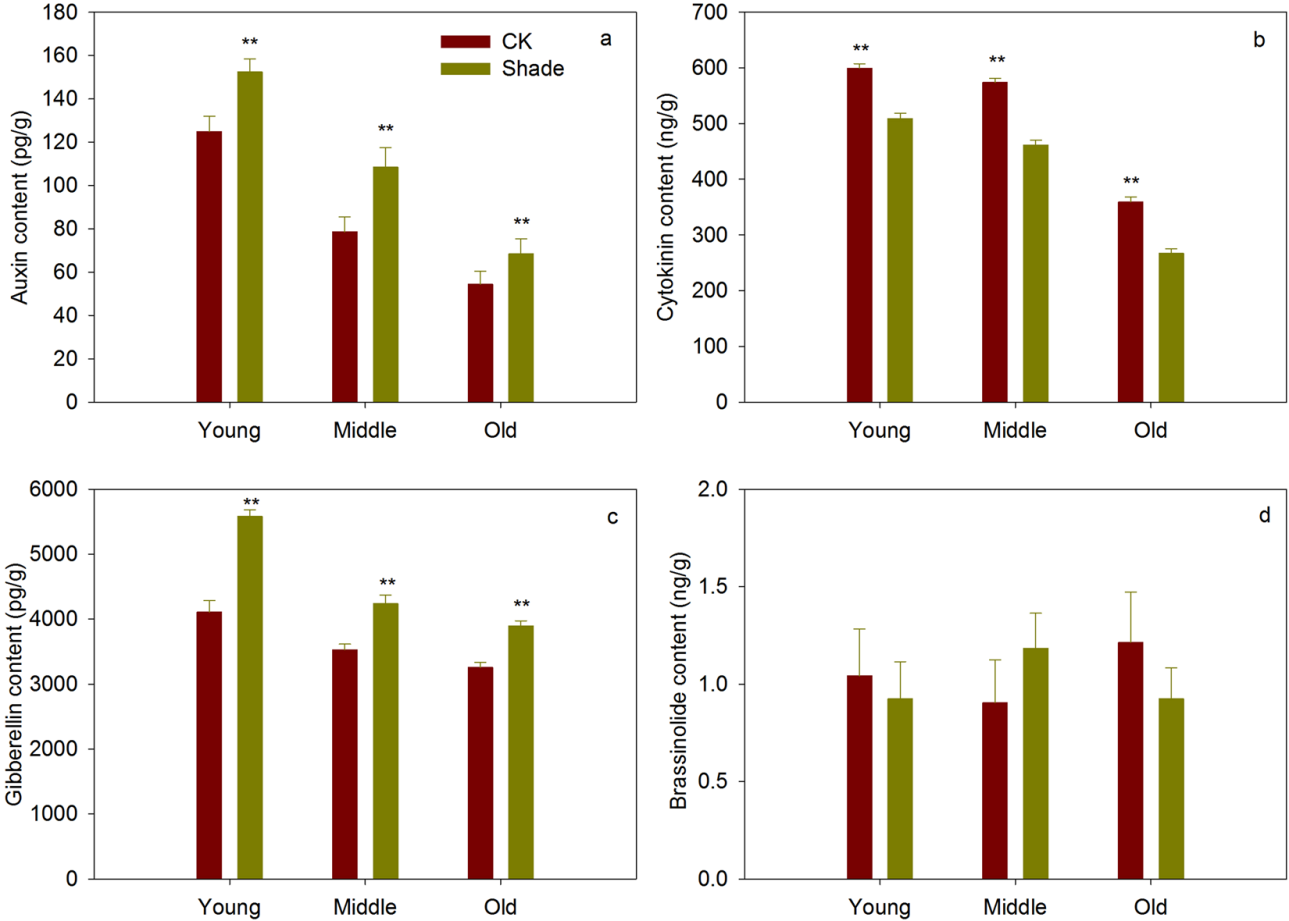
Hormones content (auxin (**a**), cytokinin (**b**), gibberellins (**c**), and brassinolide (**d**)) of soybean planted in shade and full sunlight (CK). Values are means ± SD (n = 6). Statistical significance assessed by Duncan’s t-test. * and ** indicates significant at 0.05 and 0.01 probability level, respectively.

As shown in Fig. 7a–c, shade significantly increased the content of gibberellins and auxin, and significantly decreased the content of cytokinin in young, middle and old leaves. But there was no difference in brassinolide between shade plants and full sunlight plants (Fig. 7d).

## Discussion

Smaller and thinner leaves of soybean in shade is consistent with our previous study^28^, and as described in other species^14, 31–40^. Due to the reduces of cell number, our results confirmed that low light reduced leaf thickness by inhibiting anticlinal cell expansion rates, this effect on cell expansion was preceded by an effect on cell division, leading to one less layer of palisade cells^41^. Most of the studies have been generally accepted that thinner leaves have more chance to intercept light because of its low leaf dry mass per unit area (LMA)^5, 42^. But compared to thicker leaves, the thinner leaves has thinner palisade tissue and less chloroplasts, this structure is not conducive to the CO_2_ transport and dissolution^15^. Therefore, thinner leaves don’t have a strong capacity of photosynthetic and biomass accumulation. In this research, a decreased shoot biomass confirmed that. In addition, soybean exhibited increased main stem length and decreased stem diameter in shade, this might be used for searching light under shade^8, 10, 43–45^. Decreased biomass partition to the leaf was also observed when soybean grown in shade condition, which suggested that soybean invested more resource in the stem growth at the expense of the leaf expansion. Taken together, these results suggested that shade indeed inhibited leaves growth.

Leaf size mainly determined by the co-ordination of cell division and cell expansion^18–20^. In this study, shade treatment significantly decreased cell numbers in developing leaves and maturing leaves, this is consistent with previous study that the individual leaf area and epidermal cell number were both decreased under the shading conditions^22, 28^. The quantitative real-time (PCR) analysis found six down regulated genes about cell proliferation (*AN3*, *ANT*, *KLUH*, *UBP15*, *CYCD3*, *ROT4*)^46–49^ and five down regulated genes for both cell proliferation and enlargement (*ARF2*, *EBP1*, *RGA*, *DA1*, *EOD1*)^50–54^ genes in axillary bud or young leaves. Meanwhile, the shade induced change in cell number of young leaves was significantly greater than those of old leaves (Table 2), which confirmed the magnitude of the shade effect on cell proliferation depended on different leaf developmental stages, and it was more distinct in young leaves than old leaves. Previous study have also shown that when leaf reached twenty percent of its final size, the cell division will complete^55^. Therefore, our results proved that shade inhibited the cell division and reduced cell numbers mainly in young leaves.

**Table 2:** CLA, CCN and CCS of soybean planted in shade. CLA (change of leaf area) = (leaf area of shade plants)/(leaf area of full sunlight plants).

	CLA	CCN	CCS
Young	0.74 ± 0.02a	0.79 ± 0.01a	0.84 ± 0.03a
Middle	0.80 ± 0.02b	0.84 ± 0.01b	0.95 ± 0.02b
Old	0.80 ± 0.01b	0.84 ± 0.01b	0.96 ± 0.03b

CCN (change of cell number) = (cell number of shade plants)/(cell number of full sunlight plants). CCS (change of cell size) = (cell size of shade plants)/(cell size of full sunlight plants). Data are means ± SD (n = 4). Statistical significance assessed by Duncan’s t-test. Letters on each row represents a significant level of 0.01 (p < 0.01).

Plant growth also requires irreversible cell enlargement. Our measurement also found that the cell sizes in both young and old leaves were significantly decreased in shade. The quantitative real-time (PCR) analysis suggested shade inhibited cell expanding in young and middle leaves, because most of the genes about cell enlargement and both cell proliferation and enlargement were down regulated in axillary bud, young and middle leaves. It is generally known that cell wall loosening and expansion were the main reasons causing the expansion and elongation of plant cells^56, 57^, cell wall was regarded as an important regulatory point during shade avoidance^58, 59^. Some researches has reported that in shade condition, cell wall synthesis-related genes were down-regulated^28^, shading contributes to the reduction of stem mechanical strength by decreasing cell wall synthesis in Japonica Rice^60^. These results suggested cell wall biosynthesis were inhibited in shade. Thus, we speculated the decreased cell size in shade may be caused by decreased cell wall synthesis.

Auxin was thought to be an important hormone to regulate leaf development, growth, expansion and longevity^61–64^. Conversely, leaf is an important organ for auxin synthesis^65^. Many critical genes have identified involved in auxin regulation under shade^11^. It has been reported that higher concentrations of auxin result in inhibition of cell expansion and smaller leaves, only lower concentrations promote cell expansion^61^. In this study, shade significantly increased the auxin content, this is consistent with previous reports that both low photosynthetically active radiation and low red light: far red light ratio can increase auxin content in leaves^66, 67^. So, our results of smaller soybean leaves and increased auxin content in shade suggested that shade indeed suppresses leaf expansion by increase auxin content.

In addition to auxin, cytokinin also plays a central role during the cell cycle, it is involved in leaf initiation and plays an important role in SAM maintenance^68–70^. Earlier studies showed that cytokinin is associated with perception of both the duration and quantity of sunlight^71^. Carabellin *et al*. found that shade signal triggerred a rapid arrest of leaf primordium growth depends on auxin-induced cytokinin breakdown^24^. Pons also pointed that shade declined leaf expansion, but when cytokinin was applied to shaded leaves, it turned out that the leaf expansion of shaded leaf can restore to full sunlight levels^72^. In our study, cytokinin content was decreased, cell proliferation (*ANT*, *AN3*, *KLUH*, *UBP15*, *CYCD3*) and both cell proliferation and cell enlargement (*ARF2*, *EBP1*, *RGA*, *DA1*, *EOD1*) genes were down-regulated in axillary bud and young leaves, which suggested decreased cytokinin might have induced decreased cell proliferation. In addition, the shade induced change in cell number was greater than change in cell size (Table 2), combined with smaller leaves, we supposed that soybean leaves was inhibited mainly caused by decreased cell number. Many studies have found that GA and light interact in regulating hypocotyl elongation, cotyledon opening and light-responsive gene expression, their pathways seem to that GA promote growth through cell proliferation rate and cell expansion by stimulating the destruction of growth-repressing DELLA proteins^52, 73–75^. In this study, smaller leaves in shade showed significant higher GA content than leaves in full sunlight, this is consistent with recent report that low irradiance PAR has very likely induced an overall increase in GA biosynthesis^76^. But in our observation, inhibition of cell proliferation and expansion accompanied with increased GA content, this is different from previous studies^52, 73–75^. To our knowledge, these researches mainly focused on hypocotyl, stem and petiole, but the present study is focus on leaves, heterogeneity of GA function in responses to shade in leaf and petiole for this regulation is currently not well understood and needed further study.

In recent years, it has become evident that hormonal pathways determine the final outcome of the individual hormone actions usually by a complex network of interactions and feedback circuits^71, 77^. Shoot apical meristem activity was controlled by the auxin – cytokinin - gibberellin interaction^78^. The interaction of light quality and irradiance with gibberellins, cytokinins and auxin in regulating growth of *Helianthus annuus* hypocotyls^79^. In this study, our results found increased auxin and gibberllin contents and deceased cytokin in leaves (Fig. 7). Although increased auxin and gibberllin might increase the cell proliferation, but the deceased cytokinin had opposite effects. In our observation, soybean leaves was inhibited by decreased cell number in young phase, whether this results was mainly caused by reduction of cytokinin contents still need to be tested. Our study confirmed that auxin, cytokinin and gibberellin involved in regulation on leaf development, but we still don’t know which hormone is the most important factor and whether auxin – cytokinin - gibberellin interactions control leaf development of soybean in shade. Thus, our further step needs to study the auxin, cytokinin, gibberellin and their interaction on leaf expansion in shade.

## Materials and Methods

### Plant material and growth

The soybean (Nandou12, oval leaf, was widely planted in relay intercropping system in southeast of China) was employed in this study. The experiment was conducted in a greenhouse of Sichuan Agricultural University.

Five to nine soybean seeds were sown in plastic pots (30 cm-diameter, 20 cm-height) and watered to maintain the soil at field capacity. Seeds germinated and grown for 30 days under shade (shaded conditions were provided by a covering of green filters (Q-MAX 122, USA), 25% of full sunlight, R: FR (0.5~0.6)), or full sunlight conditions as control (CK). After 30 days, ten plants randomly selected from ten pots were tagged for sampling. From eight o’clock to nine o’clock in the morning, six middle trifoliate of leaves in positions 1–3 were sampled (1 were fully expanded mature leaves, 2 were incomplete maturation leaves, 3 were the youngest expanding leaves, respectively), from six individual plant, then wrapped with foil, and frozen in liquid nitrogen immediately and stored at −80 °C until for RNA extraction and hormone analysis.

### Morphology and growth measurements

Other four plants were used to measure the stem length, stem diameter and petiole length. Leaves were scanned using a flatbed scanner (CanoScan LiDE 200, Canon Inc., Japan), and the leaf area (LA, cm^2^) was measured by Image J 1.45 s. Afterwards, the leaves, petioles, and stems were exposed to 105 °C for 0.5 h and then dried to a constant weight at 80 °C to separately determine their biomasses.

### Microscopic observations

Three middle segments of leaves (5 mm × 5 mm) avoiding midrib were sampled and fixed in formalin–acetic acid–alcohol (FAA) solution used for paraffin. After the capture of microphotographs, total leaf thickness, cell length and cell width were measured by Image J 1.45 s. The cell size and the density of palisade cells per unit area in the subepidermal layer was determined. Then the density was multiplied by the leaf area to calculate the total palisade cell number of leaf. This determination was repeated on six field of view.

### Gene expression analysis

We selected twenty genes involves in leaf development and growth, BLASTed against known Arabidopsis genes in phytozome v12.0 to find the homologues in soybean, and then designed primers to assess the expression levels of these genes. The soybean homologues information and primers used in the qPCR are provided in supplementary materials (supplementary Table S1). RNA was extracted following manufacturer’s protocol (Invitrogen, Carlsbad, CA, USA). PCR was performed with Power SYBR Green PCR Master Mix on ABI 7300 real-time PCR system (Applied Biosystems, Foster City, CA, USA) following the manufacturer’s protocol. The PCR was conducted as described by Gong *et al*.^28^. Soybean ACT11 (Glyma18g52780) was used as control^80^, Three biological replicates were conducted and three technical replicates for each sample on the same plate was performed. Expression levels of tested genes were displayed relative to a value of unity for old leaves in full sunlight.

### Plant hormones analysis

Hormones contents were determined by enzyme-linked immunosorbent assay (ELISA) according to Yang^81^. Briefly, samples (1 g dry weight [FW]) were used for extraction of plant hormones. Antibodies against IAA, GAs, CTK and BRs were used to determine hormones contents using an ELISA reader. ELISA kit was purchased from China Agricultural University. Determination was conducted according to the manufacturer’s protocol. Three biological replications were performed in analysis.

### Statistical analyses

ANOVA in SPSS software (SPSS, Chicago, USA) was used to analysis the differences between shade and full sunlight (control) treatments. All measured and calculated features were analyzed as dependent variable. SigmaPlot was used for all mapping.

## Electronic supplementary material

Supplementary information
