# Supplementary material for: Shade Inhibits Leaf Size by Controlling Cell Proliferation and Enlargement in Soybean
Source: Sci Rep. 2017 Aug 23;7:9259. doi: 10.1038/s41598-017-10026-5 (PMC5569092; doi:10.1038/s41598-017-10026-5)
Supplement: Supplementary file 1 — Supplementary information [file 41598_2017_10026_MOESM1_ESM.doc]

**Title page**

**The full title:** Shade Inhibits Leaf Size by Controlling Cell Proliferation and Enlargement in Soybean

**Author list:** Yushan Wu ^a, b, c, †^, Wanzhuo Gong ^d, †^, Wenyu Yang ^a, b, c^*^*^*

1. College of Agronomy, Sichuan Agricultural University, Chengdu 611130, P.R. China;

b. Key Laboratory of Crop Ecophysiology and Farming System in Southwest, Ministry of Agriculture, Chengdu 611130, P.R. China

c. Sichuan Engineering Research Center for Crop Strip Intercropping System, Chengdu 611130, PR China

d. Characteristic Crops Research Institute, Chongqing Academy of Agricultural Sciences, Chongqing, 402160, P.R. China

† These authors contributed equally to this work

^*^Corresponding author: E-mail: [mssiyangwy@sicau.edu.cn](mailto:mssiyangwy@sicau.edu.cn)

**Supplementary**

Fig S1 Shoot biomass of soybean seedlings planted in shade and full sunlight (CK). Values are means ±SD (n = 4). Statistical significance assessed by Duncan’s t-test. ** indicates significant at 0.01 probability level


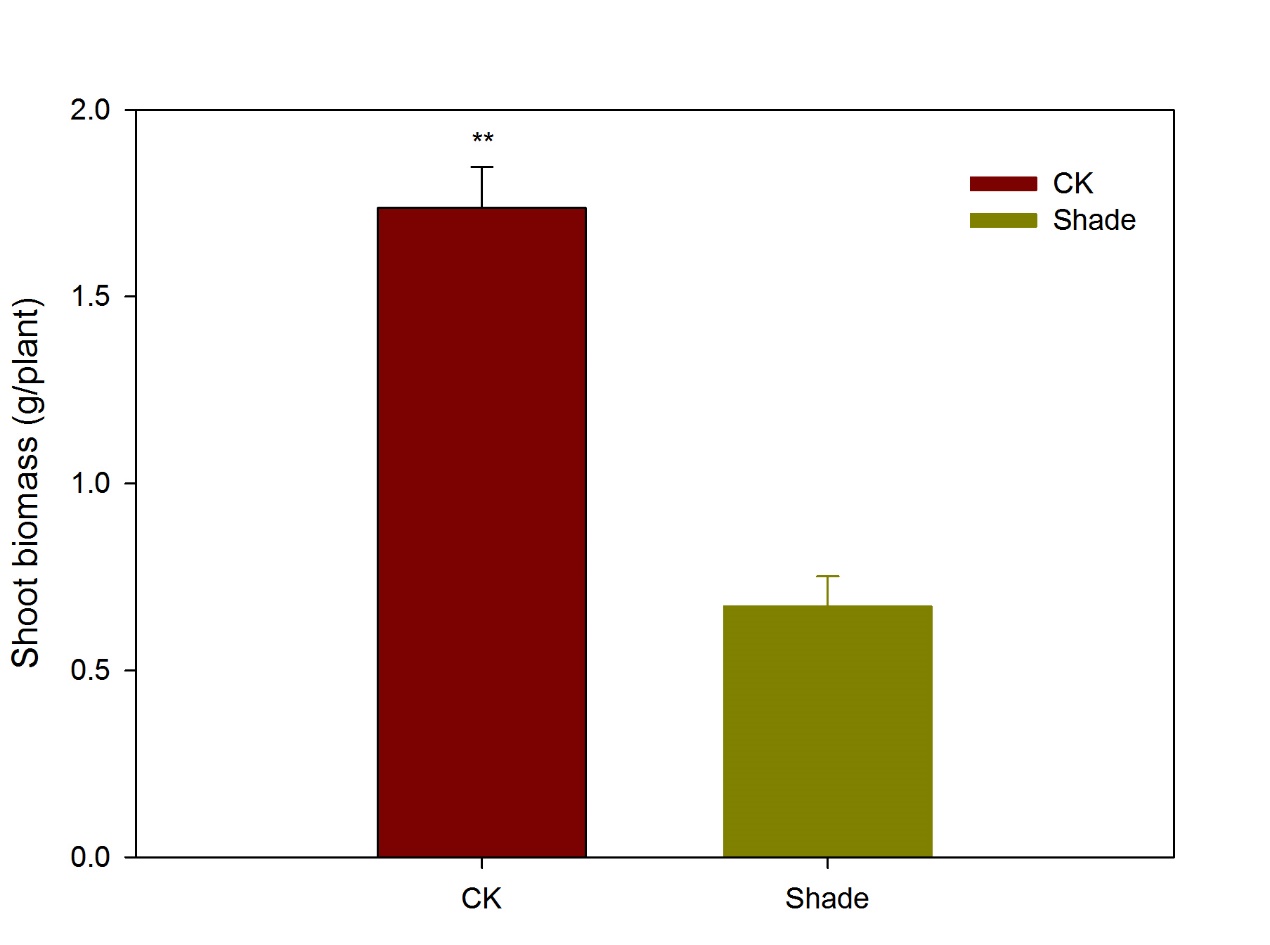


Table S1. Information and primers of soybean homologues involves in leaf development and growth according to Arabidopsis gene models

| Function | Gene | Arabidopsis model | Soybean model | qPCR-Primers |
| --- | --- | --- | --- | --- |
| **Primordium size** | SWP | AT3G04740.1 | Glyma.19G245100.1 | TTATCCGACACCGACAAGAAGA  TGGCGGAAGGTACATCGTAAA |
| **Cell proliferation** | ANT | AT4G37750.1 | Glyma.05G108600.1 | TGGGACAACAGCTGCAAGAA  TCCATGTTGGTGGTGTCTTGTT |
|  | AN3/AtGIF1 | AT5G28640.1 | Glyma.10G164100.1 | TCCTCGCTCCTGTACTCCCAA  CTCCTCCGGTTCCTATGGTTG |
|  | GRF5 | AT3G13960.1 | Glyma.07G038400.1 | TTCCAACTCCAGAATCCAAACC  CCCTACATAACGCAATAACATCACA |
|  | KLUH | AT1G13710.1 | Glyma.02G119600.1 | GGATTTGCAGGGTGTGAGGA  GAGAACCATCCGAGCCAGAG |
|  | UBP15 | AT1G17110.2 | Glyma.15G248200.1 | TTGTCCCAGAGCCATCACCTA  ACTCACGCCTGACGTTTTCG |
|  | CYCD3 | AT4G34160.1 | Glyma.20G126700.1 | ATGGTGGGATGGAATACAAAACT  GCTGCTTGGGATTTGCTCATAC |
|  | JAG | AT1G68480.1 | Glyma.10G273800.1 | AGTTGACGAGCCTGGTTCAG  CGGTACCATGAGACCAGAACG |
|  | ROT4 | AT2G36985.1 | Glyma.03G182700.1 | CTTTATGGTGCCAAATCAGGG  GCAACGGCGAAGAATGTAGAA |
|  | ARGOS | AT3G59900.1 | Glyma.07G274000.1 | CCACCAGTCCCAAGGAAGAT  TAGAAGGCATAAAAGCCGAGAT |
| **Cell enlargement** | EXP10 | AT1G26770.2 | Glyma.20G033900.1 | CCTCCACCATAGGCATGAGC  ACTCTGCTTCACTTCACACCC |
|  | TOR | AT1G50030.1 | Glyma.11G002600.1 | TGACAAAGTTCGGCAGCATAGT  GCTCACCAAAATACAAACGGCT |
|  | ROT3 | AT4G36380.1 | Glyma.11G073300.1 | TACAACTACGAGACGGTGCG  ACCGACTGAAATGGAAGCGT |
|  | SAUR19 | AT5G18010.1 | Glyma.04G006700.1 | TGGTTTGGTCCTCTATCGCAT  TTTGTGTGTTTCGCTGCTGTT |
| **Both cell proliferation and enlargement** | ARF2 | AT5G62000.1 | Glyma.06G164900.1 | CGGCATTCTCATCTTGGTTTG  CAAGATCCTTTGTCGGGTCA |
|  | EBP1 | AT3G51800.2 | Glyma.03G055500.1 | CAACCCTTGTATCTGGATTGGAT  AGAAGATTGCCGCAGCGTAT |
|  | RGA | AT2G01570.1 | Glyma.11G216500.1 | GTCACCGCCTGCGAATAGAG  GGTAGAGCGTCACGAGACTTTG |
|  | DA1 | AT1G19270.1 | Glyma.14G077800.1 | TCTCAGAGGAGCTCAGCACT  TAACCTTTAAGCCGCAGCCA |
|  | EOD1 | AT3G63530.1 | Glyma.12G237700.1 | ACGCAGGGTTTCCTTATGCT  CTACCAGGGCCAGACAATCC |
| **Meristemoid division** | PPD2 | AT4G14720.1 | Glyma.20G150000.1 | CCCATTTGAGGCATGGACCT  GTAGAGGGTCAGGCAAGCAG |
| **Reference** | ACT11 | AT3G12110.1 | Glyma.18G290800.1 | ATCTTGACTGAGCGTGGTTATTCC  GCTGGTCCTGGCTGTCTCC |
